# Left ventricular mass regression, all-cause and cardiovascular mortality in chronic kidney disease: a meta-analysis

**DOI:** 10.1186/s12882-022-02666-1

**Published:** 2022-01-16

**Authors:** Kevin C. Maki, Meredith L. Wilcox, Mary R. Dicklin, Rahul Kakkar, Michael H. Davidson

**Affiliations:** 1 Department of Applied Health Science, Indiana University School of Public Health, 1025 E 7th St #111, Bloomington, IN 47405 USA; 2 Midwest Biomedical Research, Addison, IL USA; 3 Brigham and Women’s Hospital, Boston, MA USA; 4 University of Chicago Pritzker School of Medicine, Chicago, IL USA

**Keywords:** Chronic kidney disease, Cardiovascular disease, Left ventricular mass index, Mortality

## Abstract

**Background:**

Cardiovascular disease is an important driver of the increased mortality associated with chronic kidney disease (CKD). Higher left ventricular mass (LVM) predicts increased risk of adverse cardiovascular outcomes and total mortality, but previous reviews have shown no clear association between intervention-induced LVM change and all-cause or cardiovascular mortality in CKD.

**Methods:**

The primary objective of this meta-analysis was to investigate whether treatment-induced reductions in LVM over periods ≥12 months were associated with all-cause mortality in patients with CKD. Cardiovascular mortality was investigated as a secondary outcome. Measures of association in the form of relative risks (RRs) with associated variability and precision (95% confidence intervals [CIs]) were extracted directly from each study, when reported, or were calculated based on the published data, if possible, and pooled RR estimates were determined.

**Results:**

The meta-analysis included 42 trials with duration ≥12 months: 6 of erythropoietin stimulating agents treating to higher vs. lower hemoglobin targets, 10 of renin-angiotensin-aldosterone system inhibitors vs. placebo or another blood pressure lowering agent, 14 of modified hemodialysis regimens, and 12 of other types of interventions. All-cause mortality was reported in 121/2584 (4.86%) subjects in intervention groups and 168/2606 (6.45%) subjects in control groups. The pooled RR estimate of the 27 trials ≥12 months with ≥1 event in ≥1 group was 0.72 (95% CI 0.57 to 0.90, *p* = 0.005), with little heterogeneity across studies. Directionalities of the associations in intervention subgroups were the same. Sensitivity analyses of ≥6 months (34 trials), ≥9 months (29 trials), and >12 months (10 trials), and including studies with no events in either group, demonstrated similar risk reductions to the primary analysis. The point estimate for cardiovascular mortality was similar to all-cause mortality, but not statistically significant: RR 0.67, 95% CI 0.39 to 1.16.

**Conclusions:**

These results suggest that LVM regression may be a useful surrogate marker for benefits of interventions intended to reduce mortality risk in patients with CKD.

**Supplementary Information:**

The online version contains supplementary material available at 10.1186/s12882-022-02666-1.

## Background

Chronic kidney disease (CKD) is a major public health issue, with an estimated global prevalence of 13.4% (broadly ranging from approximately 5 to 15% across the world); approximately 5-7 million persons worldwide have end-stage kidney disease (ESKD) requiring dialysis, and, ultimately, renal replacement therapy [1]. Mortality is increased in patients with CKD compared to those without CKD, and an important driver of mortality is increased risk for cardiovascular disease (CVD) [2, 3]. Left ventricular hypertrophy (LVH) is present in 15-21% of the general population, but affects 50-70% of patients with CKD, and as many as 80% of patients with CKD on dialysis [4–8]. LVH is a marker for the hemodynamic (afterload and preload) and non-hemodynamic (humoral, endocrine, autonomic and cellular) changes that impact the myocardium in CKD. Higher left ventricular mass (LVM) or left ventricular mass index (LVMI) predicts increased risk of adverse cardiovascular outcomes and total mortality in several patient populations [9–16], including ESKD patients [17–20]. The investigators of a previous systematic review and meta-regression analysis of 73 randomized controlled trials (RCTs) that investigated the validity of LVM regression as a surrogate endpoint for all-cause and cardiovascular mortality in CKD concluded that there was no clear or consistent association between intervention induced LVM change and mortality [21]. However, a majority of the interventions evaluated in the included trials had little or no effect on LVM, and some actually produced an increase. Moreover, the analysis included studies with durations as short as 3 months, which may not be a sufficient time for benefits to become apparent [10]. The primary objective of the present meta-analysis was to address the question of whether treatment-induced reductions in LVM were associated with all-cause mortality in patients with CKD over periods ≥12 months. Cardiovascular mortality was evaluated as a secondary outcome.

## Methods

This investigation was performed in accordance with the recommendations of the Preferred Reporting Items for Systematic Reviews and Meta-Analyses: The PRISMA Statement [22] and the Methodological Standards for Meta-Analyses and Qualitative Systematic Review of Cardiac Prevention and Treatment Studies: A Scientific Statement from the American Heart Association [23]. The meta-analysis protocol was registered with the international prospective register of systematic reviews (PROSPERO registration number CRD42018106425).

The aforementioned systematic review and meta-analysis publication by Badve et al. included a list of the characteristics and outcomes of studies that had been evaluated for possible inclusion [21]. That list of studies was re-evaluated for possible inclusion in the present meta-analysis and an additional literature search was conducted using the PubMed database, to search for other qualifying papers, particularly those published since the cutpoint specified by Badve et al. (December 2015) through December 2020. The search criteria included: 1) randomized trials that reported treatment effects on LVM in adults or children with any stage of CKD; and 2) randomized trials that reported treatment effects on LVM in adults or children in the general population that included a separate subgroup analysis of participants with CKD. The study exclusion criteria included: 1) observational studies; 2) trials with follow-up duration <6 months; and 3) trials involving kidney transplant recipients. The search terms used are listed in Supplemental Table 1. The literature search was updated and expanded to other search engines in October 2021 by searching the Cochrane Central Register of Controlled Trials for articles published from 2015-2021 that met the search criteria.

Because the objective of the present analysis was to address the question of whether treatment-induced reductions in LVM were associated with all-cause and cardiovascular mortality in patients with CKD, the primary analysis of the study was further limited to studies in which the intervention had a regressive effect on LVM. Specifically, the primary analysis included studies in which the mean change in standardized LVM (usually expressed as LVMI) was ≤ −0.01 standard deviations (SD) for the intervention group change minus the control group change. The primary analysis was also limited to studies that followed subjects for a period adequate to collect mortality events, i.e., ≥12 months. This threshold was chosen because the echocardiographic sub-study results from the Losartan Intervention for Endpoint Reduction in Hypertension (LIFE) trial did not show clear evidence of separation between treatments during the first several months [10]. Results are also reported for other follow-up time frames in sensitivity analyses. In cases where multiple secondary publications of the same data set were identified, the publication with the most complete data was used, and additional data from secondary sources were extracted. Also, only data from the first phase of randomized crossover trials were eligible.

Qualitative information and quantitative data were extracted from each publication meeting the inclusion criteria. All data from eligible trials were abstracted by an independent scientist and reviewed by the lead investigator (KCM). Any discrepancies were resolved by discussion with an additional scientist and by referencing the original publication.

Measures of association in the form of relative risks (RRs) with associated variability and precision (95% confidence intervals [CIs]) were extracted directly from each study, when reported, or were calculated based on the published data, if possible. Pooled RR estimates for the meta-analysis were completed using Comprehensive Meta-Analysis, Version 3 (Biostat, Englewood, NJ). When a study had 0 events in 1 group, the software added 0.5 to the number of events and non-events for each group for computation of the log risk ratio and its variance. Statistical significance for individual studies and for pooled RRs was declared when the 95% CI did not include the null value of 1.0 (i.e., p-value <0.05). Studies were weighted according to the inverse of the variance of each study’s effect using random effects models. Statistical heterogeneity was assessed using Cochran’s Q and the I^2^ statistic. The Cochrane Handbook defines an I^2^ value of 0 to 40% as low heterogeneity, which “might not be important” [24]. An I^2^ value of ≥40% was used to designate moderate or higher heterogeneity. Comprehensive Meta-Analysis uses the inverse variance method for calculating the weighted pooled RR under the fixed effect models, which were considered secondary [25].

The primary outcome variable was RR for all-cause mortality between intervention and control groups for trials with ≥12 months of treatment in which any reduction in mean LVM was reported (whether or not statistically significant in each individual trial). Thus, the exposure of interest was not the treatment applied, but rather the presence of treatment induced LVM regression relative to the control condition. RR for cardiovascular mortality was similarly evaluated as a secondary outcome. Sensitivity analyses were conducted to assess minimum follow-up thresholds of ≥6, ≥9, and > 12 months. Sensitivity analyses were also conducted including studies in which 0 events were observed in both comparison groups (by summing totals as if all subjects were in the same trial) to illustrate the degree to which such “no event” trials may have influenced the results. Subgroup analyses by type of intervention (erythropoietin stimulating agent [ESA], renin-angiotensin-aldosterone system inhibitor [RAASi], non-conventional hemodialysis [NCHD], and other), according to LVM or LVMI change > or ≤ the median, according to whether the studies enrolled adults only or may have also enrolled children, and according to whether the studies exclusively (or predominantly) enrolled patients with ESKD or did not include patients with ESKD, were also completed for all-cause mortality.

A post-hoc random effects meta-regression was performed to investigate the effect of mean change in standardized LVM on the effect size for all-cause mortality and for cardiovascular mortality. Regression coefficients and the percentage of between-study heterogeneity explained by the mean change in standardized LVM (R^2^_*_) were generated using Comprehensive Meta-Analysis, Version 3 (Biostat, Englewood, NJ). The random effects variance components of the models were computed using method of moments. Sensitivity analyses were also completed using maximum likelihood and restricted maximum likelihood, which did not alter the results materially.

The presence of publication bias was assessed visually by examining funnel plots measuring the standard error as a function of effect size, as well as statistically by using Egger’s regression method [26, 27]. Study quality was assessed using the Grading of Recommendations Assessment, Development and Evaluation (GRADE) method [28].

## Results

A description of the results of the literature search and study screening is shown in Supplemental Fig. 1. A total of 58 studies met the overall inclusion criteria (described in detail in Supplemental Table 2) [29–86]. As shown in Table 1, 42 studies met the inclusion criteria for the primary evaluation of the effects of LVM regression on all-cause mortality with ≥12 months of follow-up. This included 6 studies using ESAs to treat to a higher vs. lower hemoglobin target [37, 38, 43–46], 10 studies of RAASi vs. placebo or vs. another blood pressure-lowering agent [29, 34–36, 40, 47, 49, 53, 54, 70], 14 studies of more intensive vs. less intensive hemodialysis (greater frequency or vs. high-flux hemodialysis or more intensive fluid management) [30, 48, 51, 56, 57, 61, 64, 67, 68, 72, 74, 76, 78, 82], and 12 studies of other types of interventions [31, 33, 52, 62, 66, 75, 77, 79, 83–86]. A total of 30 studies enrolled predominantly ESKD patients (defined as patients on hemodialysis) [29–31, 35, 36, 38, 40, 48, 49, 51, 54, 56, 57, 61, 64, 67, 68, 70, 72, 74–79, 82–86] and 12 studies did not enroll patients on dialysis [33, 34, 37, 43–47, 52, 53, 62, 66].

**Table 1:** Trials of ≥12 months follow-up in patients with chronic kidney disease in which left ventricular mass was reduced in the intervention group relative to the control group^a^

Author, Year	Intervention	Intervention		Control		F/U,	LVM ∆,
		Events	Subjects	Events	Subjects	months	SMD
Levin, 2005 [37]	ESA/higher vs. lower hemoglobin target	1	85	3	87	24	−0.20
Parfrey, 2005 [38]	ESA/higher vs. lower hemoglobin target	13	296	20	300	24	−0.12
Macdougall, 2007 [43]	ESA/higher vs. lower hemoglobin target	1	65	6	132	36	−0.25
Ritz, 2007 [44]	ESA/higher vs. lower hemoglobin target	0	89	0	83	15	−0.05
Pappas, 2008 [46]	ESA/higher vs. lower hemoglobin target	1	15	3	16	12	−0.97
Cianciaruso, 2008 [45]	ESA/higher vs. lower hemoglobin target	1	46	0	49	24	−0.07
Suzuki, 2002 [34]	RAASi vs. placebo or standard treatment						
	without LVH, 5 mg benazepril	0	12	0	12	12	−0.52
	without LVH, 2.5 mg benazepril	0	12	0	12	12	−0.43
	with LVH, 5 mg benazepril	0	12	0	12	12	−0.44
	with LVH, 2.5 mg benazepril	0	12	0	12	12	−0.15
London, 1994 [29]	RAASi vs. placebo or standard treatment	0	16	0	16	12	−0.57
Suzuki, 2003 [35]	RAASi vs. placebo or standard treatment	0	14	0	10	12	−0.61
Kanno, 2004 [36]	RAASi vs. placebo or standard treatment	0	12	0	12	12	−1.05
Yu, 2006 [40]	RAASi vs. placebo or standard treatment	1	24	0	22	12	−0.51
Mitsuhashi, 2009 [49]	RAASi vs. placebo or standard treatment	0	20	0	20	12	−0.89
Zeltner, 2008 [47]	RAASi vs. placebo or standard treatment	0	23	0	23	36	−0.01
Yilmaz, 2010 [54]	RAASi vs. placebo or standard treatment	0	56	1	56	12	−0.18
Ito, 2014 [70]	RAASi vs. placebo or standard treatment	2	78	5	80	24	−0.34
Ulusoy, 2010 [53]	RAASi vs. other RAASi	0	19	0	13	12	−0.49
Schrander-vd Meer, 1999 [30]	Convective HD vs. standard HD	0	12	0	12	12	−0.69
Alvestrand, 2011 [56]	Convective HD vs. standard HD	2	27	3	21	24	−0.31
Ohtake, 2012 [64]	Convective HD vs. standard HD	0	13	0	9	12	−0.26
Mostovaya, 2014 [72]	Convective HD vs. standard HD	41	358	51	356	12	−0.11
Katopodis, 2009 [48]	≥4x vs. <4x/week HD	0	9	0	9	12	−0.25
Chertow, 2010 [51]	≥4x vs. <4x/week HD	5	125	9	120	12	−0.26
Rocco, 2011 [61]	≥4x vs. <4x/week HD	2	45	1	42	12	−0.20
Chen, 2011 [57]	HD + hemoperfusion vs. HD	6	51	14	49	24	−5.50
Hur, 2013 [67]	Fluid management vs. standard HD	2	78	4	78	12	−0.39
Whalley, 2013 [68]	Early vs. later dialysis initiation	2	91	1	91	12	−0.20
Odudu, 2015 [74]	Individual dialysate cooling vs. standard	2	36	1	37	12	−0.34
Liu, 2016 [76]	Reduced dialysate sodium	3	32	2	32	12	−0.55
Marshall, 2020 [82]	Reduced dialysate sodium	2	49	1	50	12	−0.15
Jardine, 2017 [78]	Extended HD vs. standard	5	100	2	100	12	−0.19
Howden, 2013 [66]	Exercise vs. usual activity	0	41	0	42	12	−0.06
Schrier, 2002 [33]	Lower vs. higher blood pressure target	1	42	1	37	84	−0.92
Nakamura, 2002 [31]	Dilazep vs. placebo	0	20	0	20	12	−0.23
Hotu, 2010 [52]	Nurse/community vs. physician/clinic	2	33	0	32	12	−0.69
Zamboli, 2011 [62]	Furosemide vs. no furosemide	0	20	0	20	12	−0.55
Higuchi, 2016 [75]	Levocarnitine vs. no levocarnitine	5	110	7	112	12	−0.40
Lin, 2016 [77]	Spironolactone vs. placebo	12	125	25	128	24	−0.55
Miskulin, 2018 [79]	Lower vs. higher blood pressure target	4	62	1	64	12	−0.05
Fujii, 2018 [85]	Lanthanum carbonate vs. calcium carbonate	1	50	1	55	18	−0.07
Rutherford, 2021 [86]	Allopurinol vs. placebo	2	40	5	40	12	−0.10
Dorr, 2021 [84]	Etelcalcitide vs. alfacalcidol	2	32	1	30	12	−0.40
Edwards, 2021 [83]	Spironolactone vs. chlorthalidone	0	77	0	77	40	−0.12
**Total or Median**^**a**^		**121**	**2584**	**168**	**2606**	**12**	**−0.285**

∆ change, *ESA* erythropoietin-stimulating agent, *F/U* follow-up, *HD* hemodialysis, *LVH* left ventricular hypertrophy, *LVM* left ventricular mass, *RAASi* renin-angiotensin-aldosterone system inhibitor, *SMD* standardized mean difference

^a^F/U and LVM ∆ SMD are medians, others are numbers

**Fig. 1:**
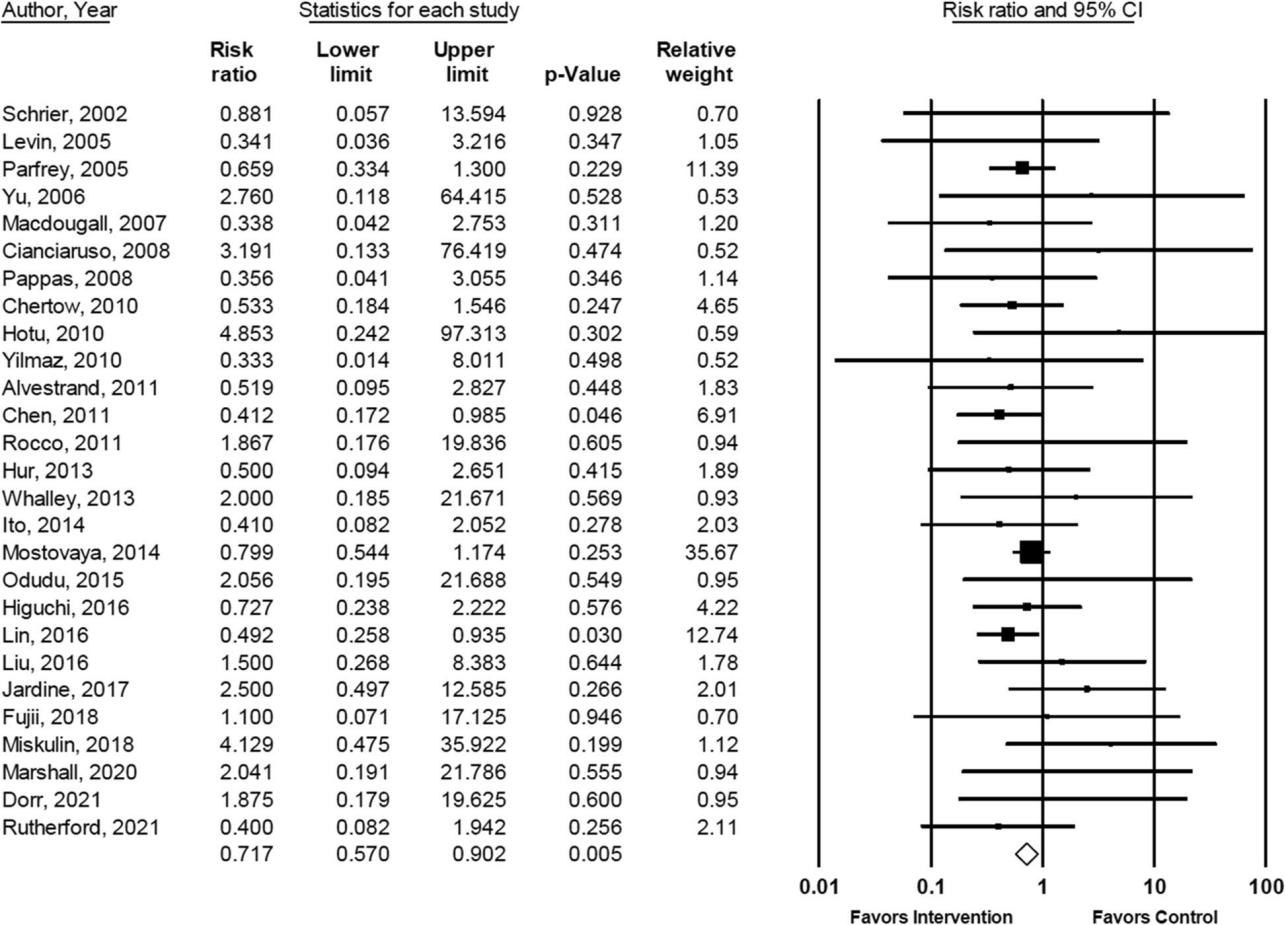
Forest plot of all-cause mortality in patients with chronic kidney disease in trials of ≥12 months follow-up in which left ventricular mass was reduced in the intervention group relative to the control group [33, 37, 38, 40, 43, 45, 46, 51, 52, 54, 56, 57, 61, 67, 68, 70, 72, 74–79, 82, 84–86]. Squares represent the relative risk (RR) of the individual studies and horizontal lines represent the 95% confidence intervals (CIs). The size of the square reflects the weight of the corresponding study in the meta-analysis. The diamonds represent the pooled relative risk of the overall effect

A total of 289 deaths were reported in 27 of the 42 qualifying trials: 121/2584 (4.86%) subjects in the intervention groups and 168/2606 (6.45%) subjects in the control groups (Table 1). The pooled RR (95% CI) estimate from the primary analysis (all-cause mortality in patients with CKD in trials of at least 12 months follow-up in which LVM was reduced in the intervention group relative to the control group and there was at least 1 event in 1 group) was 0.72 (0.57 to 0.90, *p* = 0.005) (Table 2 and Fig. 1). There was little heterogeneity across studies (I^2^ = 0.0%, Q = 18.6, p for heterogeneity = 0.851). Findings from the sensitivity analysis to evaluate the degree to which exclusion of trials for which no mortality events were reported may have affected the results were not materially different; RR = 0.73 (95% CI 0.58 to 0.91, *p* = 0.006). Results of the sensitivity analyses for the pooled RR of all-cause mortality using different thresholds of minimum follow-up time are shown in Table 3. The pooled RR for studies with >12 months follow-up showed 47% lower cumulative mortality incidence. All timeframes for minimum follow-up thresholds had 95% CIs that did not cross the null value. The median reductions in LVM in the intervention groups compared with the control groups in the categories of ≥6 (34 trials), ≥9 (29 trials), ≥12 (27 trials) and > 12 months (10 trials) were − 0.23, −0.25, −0.26 and − 0.28 standard deviations, respectively.

**Table 2:** Relative risk of all-cause mortality in patients with chronic kidney disease in trials of ≥12 months follow-up in which left ventricular mass was reduced in the intervention group relative to the control group and there was ≥1 event in ≥1 group

Author, Year	N	RR	95% CI		% Weight
			Lower	Upper	
Schrier, 2002 [33]	79	0.881	0.057	13.594	0.70
Levin, 2005 [37]	172	0.341	0.036	3.216	1.05
Parfrey, 2005 [38]	596	0.659	0.334	1.300	11.39
Yu, 2006 [40]	46	2.760	0.118	64.415	0.53
Macdougall, 2007 [43]	197	0.338	0.042	2.753	1.20
Cianciaruso, 2008 [45]	95	3.191	0.133	76.419	0.52
Pappas, 2008 [46]	31	0.356	0.041	3.055	1.14
Chertow, 2010 [51]	245	0.533	0.184	1.546	4.65
Hotu, 2010 [52]	65	4.853	0.242	97.313	0.59
Yilmaz, 2010 [54]	112	0.333	0.014	8.011	0.52
Alvestrand, 2011 [56]	48	0.519	0.095	2.827	1.83
Chen, 2011 [57]	100	0.412	0.172	0.985	6.91
Rocco, 2011 [61]	87	1.867	0.176	19.836	0.94
Hur, 2013 [67]	156	0.500	0.094	2.651	1.89
Whalley, 2013 [68]	182	2.000	0.185	21.671	0.93
Ito, 2014 [70]	158	0.410	0.082	2.052	2.03
Mostovaya, 2014 [72]	714	0.799	0.544	1.174	35.67
Odudu, 2015 [74]	73	2.056	0.195	21.688	0.95
Higuchi, 2016 [75]	222	0.727	0.238	2.222	4.22
Lin, 2016 [77]	253	0.492	0.258	0.935	12.74
Liu, 2016 [76]	64	1.500	0.268	8.383	1.78
Jardine, 2017 [78]	200	2.500	0.497	12.585	2.01
Fujii, 2018 [85]	105	1.100	0.071	17.125	0.70
Miskulin, 2018 [79]	126	4.129	0.475	35.922	1.12
Marshall, 2020 [82]	99	2.041	0.191	21.786	0.94
Rutherford, 2021 [86]	80	0.400	0.082	1.942	2.11
Dorr, 2021 [84]	62	1.875	0.179	19.625	0.95
**Pooled**^**a**^	**4367**	**0.717**	**0.570**	**0.902**	**100.00**
**Z = −2.84,** ***P*** **= 0.005, I**^**a**^ **= 0.0%, Q = 18.6, P**_**heterogeneity**_ **= 0.851**					

*CI* confidence interval, *N* number, *RR* relative risk

^a^Pooled estimates are from random effects analysis. Results from the fixed effect analyses were the same

**Table 3:** Sensitivity analyses for the pooled relative risk of all-cause mortality in patients with chronic kidney disease using different minimum follow-up thresholds for studies in which left ventricular mass was reduced in the intervention group relative to the control group and there was ≥1 event in ≥1 group^a^

Follow-up	Number of Trials	Intervention, Events/n	Control, Events/n	Pooled RR^**b**^	95% CI
≥6 Months	34	135/2541	177/2560	0.74	0.59 to 0.92
≥9 Months	29	121/2210	171/2302	0.71	0.57 to 0.90
**≥12 Months**	**27**	**121/2151**	**168/2216**	**0.72**	**0.57 to 0.90**
>12 Months	10	40/865	78/938	0.53	0.37 to 0.77

*CI* confidence interval, *RR* relative risk

^a^Pooled estimates are from random effects analysis. The category of ≥12 months of follow-up is shown in bold because it was the prespecified primary minimum follow-up threshold. Median reductions in LVM in the intervention groups compared with the control groups were − 0.23, −0.25, −0.26 and − 0.28 standardized mean differences in the categories of, ≥6, ≥9, ≥12 and > 12 months, respectively

^b^P-_heterogeneity_ = 0.923 for ≥6 months, 0.898 for ≥9 months, 0.851 for ≥12 months, and 0.971 for >12 months

Subgroup analyses according to the type of intervention, LVM or LVMI change relative to −0.285, which was the median standardized mean difference for LVM change from the analyses that included all studies, including those without events, according to enrollment of adults only or possible inclusion of children, and according to the presence or absence of ESKD are shown in Table 4. None of the subgroups according to type of intervention showed results that excluded the null with 95% confidence, but all 3 of the main categories showed pooled RR values below 1.0 (ESA RR = 0.60, RAASi RR = 0.55, and NCHD RR = 0.78). The 13 studies which had a change in LVM or LVMI ≤-0.285 had a RR (95% CI) of 0.60 (0.41 to 0.88), whereas the 14 studies with a change > − 0.285 had a RR (95% CI) of 0.80 (0.60 to 1.06) (Q = 1.377; p for heterogeneity between the point estimates for the subgroups = 0.241). The 23 studies that enrolled only adult subjects had a RR (95% CI) of 0.71 (0.56 to 0.91), whereas the 4 studies that enrolled children and adults (*n* = 2) or did not report the minimum subject age (*n* = 2) had a RR (95% CI) of 0.77 (0.34 to 1.78) (Q = 0.028; p for heterogeneity between the point estimates for the subgroups = 0.866). The 21 studies of subjects with ESKD had a RR (95% CI) of 0.72 (0.57 to 0.91), whereas the 6 studies of subjects without ESKD had a RR (95% CI) of 0.66 (0.24 to 1.81) (Q = 0.028; p for heterogeneity between the point estimates for the subgroups = 0.868). Findings from analyses of the different minimum follow-up thresholds and subgroup analyses done by including all studies, including those without events, were similar to those which included only studies with at least 1 event in at least 1 group.

**Table 4:** Subgroup analyses by type of intervention, according to LVM or LVMI change relative to the median, according to whether the study possibly included children, and according to the presence or absence of ESKD for the pooled relative risk of all-cause mortality in patients with chronic kidney disease in studies with ≥12 months of follow-up in which left ventricular mass was reduced in the intervention group relative to the control group and there was ≥1 event in ≥1 group

Intervention/ degree of LVM(I) ∆/ ESKD status/or Age, number of studies	Intervention Events/subjects	Control Events/subjects	Median F/U, LVM ∆^**a**^	Pooled RR^**b**^	95% CI
ESA, 5	17/507	32/584	24, −0.20	0.60	0.34 to 1.08
RAASi, 3	3/158	6/158	12, −0.34	0.55	0.15 to 2.03
NCHD^c^, 11	72/992	89/976	12, −0.26	0.78	0.58 to 1.06
Other, 8	29/494	41/498	12, −0.40	0.67	0.42 to 1.08
> −0.285^d^, 14	80/1468	102/1542	12, −0.17	0.80	0.60 to 1.06
≤ −0.285, 13	41/683	66/674	12, −0.51	0.60	0.41 to 0.88
Adults only, 23	111/1902	155/1971	12, −0.25	0.71	0.56 to 0.91
Included children/unclear, 4	10/249	13/245	12, −0.30	0.77	0.34 to 1.78
ESKD, 21	114/1865	155/1863	12, −0.26	0.72	0.57 to 0.92
Non-ESKD, 6	7/286	13/353	24, −0.47	0.66	0.24 to 1.81

∆ change, *CI* confidence interval, *ESA* erythropoietin stimulating agent/higher vs. lower hemoglobin target, *ESKD* end stage kidney disease, *F/U* follow-up, *LVM* left ventricular mass, *NCHD* non-conventional vs. conventional hemodialysis, *RAASi* renin-angiotensin-aldosterone system inhibitor vs. placebo or standard treatment, *RR* relative risk

^a^Units for F/U are months and for LVM ∆ are standardized mean differences; results are for random effects analysis

^b^P-_heterogeneity_ = 0.828 for intervention, 0.241 for LVM(I) **∆**, 0.868 for ESKD status, and 0.866 for age

^c^NCHD includes convective hemodialysis, more frequent hemodialysis (≥4x per week), earlier start to hemodialysis, hemodialysis with hemoperfusion, fluid management during hemodialysis, and reduced dialysate sodium

^d^The cutpoint of −0.285 is the standardized mean difference from the sensitivity analysis, which included all studies with ≥12 months of follow-up, including those with no events

A total of 59 cardiovascular deaths were reported in 13 of the 42 qualifying trials: 23/2584 (0.9%) subjects in the intervention groups and 36/2606 (1.4%) subjects in the control groups. The pooled RR (95% CI) estimate from the analysis of cardiovascular mortality in patients with CKD in the 10 trials of at least 12 months follow-up in which LVM was reduced in the intervention group relative to the control group and there was at least 1 event in 1 group was 0.67 (0.39 to 1.16, *p* = 0.156). There was little heterogeneity across studies (I^2^ = 0.0%, Q = 7.7, p for heterogeneity = 0.562). The pooled RR for trials with ≥6 months (13 trials) and ≥ 9 months (11 trials) follow-up thresholds were identical to the ≥12 months analysis, but the pooled RR for the three studies with >12 months of follow-up showed 58% lower cumulative cardiovascular mortality incidence (*p* = 0.013).

Mean change in standardized LVM was not a significant predictor of the effect size for all-cause mortality (β = 0.122, 95% CI = −0.048 to 0.293, *p* = 0.160; R^2^_*_ = 0.00) or cardiovascular mortality (β = 1.261, 95% CI = −1.372 to 3.894, *p* = 0.348; R^2^_*_ = 0.00).

Risk of bias assessment for each of the 58 trials with ≥6 months duration is included in Supplemental Table 3, and an assessment of the quality of evidence of these 58 trials using the GRADE approach is included in Supplemental Table 4. The mean quality of evidence for ESA treating to higher vs. lower hemoglobin targets was of moderate quality, whereas the studies for RAASi, hemodialysis and other interventions were considered to be of low- to moderate quality.

## Discussion

The results of this meta-analysis demonstrated a pooled RR estimate of 0.72 (0.57 to 0.90, *p* = 0.005), indicating 28% lower mortality in groups with treatment-induced reductions in LVM relative to control over periods of ≥12 months. It is further notable that the pooled RR for studies with >12 months (median 24 months) follow-up showed 47% lower cumulative mortality (RR 0.53, 95% CI 0.37 to 0.77). No significant heterogeneity was observed overall. None of the 3 main subgroups of intervention type showed statistically significant results individually, but the directionality of the association was the same for each intervention type, and no statistically significant heterogeneity was noted across intervention types. Studies of subjects with ESKD showed a statistically significant reduction in mortality with treatment-induced reduction in LVM, whereas studies in subjects without ESKD did not show a significant effect, although results were similar and there was no statistically significant heterogeneity. The lack of significance in the non-ESKD subgroup was likely due to the smaller number of studies and deaths that occurred in these studies. Higher LVM has been shown to predict increased mortality risk in several patient populations, including patients with hypertension without CKD. Thus, the authors do not view it as likely that changes in LVM are less clinically important in patients with pre-ESKD.

Authors of a previous meta-regression analysis concluded that there was no compelling relationship between changes in LVM and mortality [21]. However, many of the studies included in that analysis were limited by short duration of follow-up and minimal differences between treatment arms for changes in LVM, with some of the interventions producing an increase in LVM. The present meta-analysis avoided those limitations by examining only studies that showed some degree of relative LVM reduction in studies of ≥12 months duration. However, the various interventions investigated in the included studies did not always produce reductions in LVM. Furthermore, in clinical practice, several interventions are often used simultaneously, which will result in a combined effect on LVM. The results from this meta-analysis suggest that a reduction in LVM should be considered a favorable clinical result, although prospective trials are needed to assess the use of LVM as a surrogate marker. A post-hoc meta-regression analysis indicated that degree of change in standardized LVM was not a significant predictor of the effect size for all-cause or cardiovascular mortality. However, the power of this analysis is diminished due to the restricted range of observed changes in LVM. Thus, the authors do not believe that this finding detracts from the main finding that regression of LVM was associated with reduced mortality.

The results from the LIFE study demonstrated that reversion from LVH to normal LVMI induced by antihypertensive therapy did not produce material reductions in adverse outcomes for several months [10]. Each SD reduction in LVMI was associated with a reduction of 26% (95% CI 7 to 41%, *p* = 0.008) in all-cause mortality and a reduction of 34% (95% CI 10 to 51%, *p* = 0.009) in cardiovascular mortality over the full study period in models adjusted for several covariates. Based on results from the LIFE study, the median reduction of 0.285 SD in the present meta-analysis would have been predicted to produce a ~ 8% reduction in all-cause mortality. The larger mortality reductions observed of 28% in studies with ≥12 months of follow-up, and 47% in studies with >12 months of follow-up, might be attributable to markedly higher average mortality risk in the studies included in the analysis: 6.5% cumulative mortality in the control conditions over a median follow-up period of 1 year, compared to approximately 1.4% per year over a median follow-up period of 4.8 years in LIFE [10].

In patients with CKD, total mortality is increased due to several types of events, but CVD mortality accounts for a large proportion of deaths, exceeding 50% in those with end-stage disease [17–20]. In prior studies, such as LIFE, there was a strong concordance between the effects of treatment-induced changes in LVM and results for total and cardiovascular mortality [6, 10, 87]. In the present investigation, the result for cardiovascular mortality was not statistically significant in the main analysis that included studies of at least 12 months of follow-up, but the pattern and point estimates were similar to those for the analysis of all-cause mortality, suggesting that the lack of statistical significance may have been due to insufficient statistical power.

CKD and CVD share several risk factors, such as hypertension, vascular stiffness, and endothelial dysfunction [88]. LVH is highly prevalent in CKD patients and is associated with risk of mortality and unfavorable prognosis [7, 89, 90]. In addition, CKD patients show progressively increasing LVM with decreased renal function [90]. Increased CVD mortality in CKD results from higher incidence of atherosclerotic and non-atherosclerotic (particularly arrhythmic) cardiovascular events [19, 20, 91–93]. LVM regression has been associated with reductions in both types of events [92].

LVM regression is typically associated with decreased myocardial fibrosis, which is clinically relevant because the degree of fibrosis is a strong predictor of ventricular arrhythmia risk [20]. The pathophysiologic mechanisms leading to LVH in CKD patients include additive and synergistic effects of afterload and preload factors [19]. Afterload factors, such as increased systemic arterial resistance and arterial blood pressure, and reduced large vessel compliance, lead to myocardial cell thickening and left ventricular remodeling, along with activation of the intracardiac renin-angiotensin system, which promotes cardiac fibrosis. Preload factors, including intravascular volume expansion, secondary anemia, and arteriovenous fistulas, also result in myocardial cell lengthening and left ventricular remodeling [94]. The hypertrophy of the myocardium activates cellular apoptosis and increases extracellular matrix production resulting in fibrosis [94]. Fibrosis impairs the contractility of the myocardium and causes systolic and diastolic dysfunction, potentially leading to congestive heart failure and disturbances in cardiac electrophysiology.

The present analysis was limited by the fact that patient-level data were not available, therefore only summary data could be included and the number of subjects in each type of intervention was relatively small. Because a relatively small number of studies reported cardiovascular mortality, the analysis had low statistical power for detection of an effect for this outcome. Nevertheless, the results are supportive of the conclusion that LVM regression produced by several types of interventions in patients with CKD is predictive of lower risk for all-cause mortality, which may be attributable, at least in part, to reduced cardiovascular mortality. The finding of lower all-cause mortality with LVM regression was robust in several subgroup and sensitivity analyses.

## Conclusions

Current strategies to reduce cardiovascular risk in CKD focus on control of blood pressure, fluid volume, lipids (in earlier stages), glycemic control for those with diabetes, and correction of anemia. The results from this meta-analysis show that interventions that lower LVM are associated with a significant reduction in pooled risk for all-cause mortality in CKD patients. Thus, monitoring LVM in patients with CKD may be helpful to identify those at increased risk for mortality; further, LVM change may be useful as a surrogate marker for benefits of interventions intended to reduce mortality risk in CKD.

## Supplementary Information

**Additional file 1: Table S1.** Search terms utilized for the PubMed database literature search (originally utilized by Badve et al. 2016). **Figure S1.** PRISMA flow diagram. **Table S2.** Individual study details. **Table S3.** Risk of bias assessment including studies with ≥6 months of follow-up that observed a reduction in LVM/LVMI. **Table S4.** Assessment of the quality of evidence using the GRADE approach.

## Data Availability

Not applicable.

## Abbreviations

∆: Change
CKD: Chronic kidney disease
CI: Confidence interval
ESA: Erythropoietin stimulating agent
ESKD: End stage kidney disease
F/U: Follow-up
GRADE: Grading of Recommendations Assessment, Development and Evaluation
HD: Hemodialysis
LIFE: Losartan Intervention for Endpoint Reduction in Hypertension
LVH: Left ventricular hypertrophy
LVM: Left ventricular mass
LVMI: Left ventricular mass index
N: Number
NCHD: Non-conventional hemodialysis
R^2^_*_: Percentage of between-study heterogeneity explained by the predictor in a meta-regression
RAASi: Renin-angiotensin-aldosterone system inhibitor
RCT: Randomized controlled trial
RR: Relative risk
SD: Standard deviation
SMD: Standardized mean difference
