# Supplementary material for: Left ventricular mass regression, all-cause and cardiovascular mortality in chronic kidney disease: a meta-analysis
Source: BMC Nephrol. 2022 Jan 16;23:34. doi: 10.1186/s12882-022-02666-1 (PMC8761349; doi:10.1186/s12882-022-02666-1)
Supplement: Supplementary file 1 — Additional file 1: Table S1. Search terms utilized for the PubMed database literature search (originally utilized by Badve et al. 2016). Figure S1. PRISMA flow diagram. Table S2. Individual study details. Table S3. Risk of bias assessment including studies with ≥6 months of follow-up that observed a reduction in LVM/LVMI. Table S4. Assessment of the quality of evidence using the GRADE approach. [file 12882_2022_2666_MOESM1_ESM.docx]

**Left Ventricular Mass Regression, All-cause and Cardiovascular Mortality in Chronic Kidney Disease:**

**A Meta-analysis**

**Supplementary Appendix**

Table of Contents

Table S1. Search terms utilized for the PubMed database literature search (originally utilized by Badve et al. 2016). 2

Figure S1. PRISMA flow diagram 3

Table S2. Individual study details 5

Table S3: Risk of bias assessment including studies with ≥6 months of follow-up that observed a reduction in LVM/LVMI 20

Table S4. Assessment of the quality of evidence using the GRADE approach 25

# Table S1. Search terms utilized for the PubMed database literature search (originally utilized by Badve et al. 2016).

| 1. exp Renal Dialysis/ 2. exp Kidney Failure, Chronic/ 3. exp Renal Replacement Therapy/ 4. 1 or 2 or 3 5. exp Hypertrophy, Left Ventricular/ 6. Left ventric$ hypertrophy$.tw. 7. (ventric$ adj5 hypertroph$).tw. 8. left ventricular mass index.tw 9. (ventric$ adj5 ind$).tw. 10. (ventric$ adj5 mass).tw. 11. 5 or 6 or 7 or 8 or 9 or 10 12. exp Clinical Trial/ 13. exp Controlled Clinical Trial/ 14. exp Randomized Controlled Trial/ 15. exp Random Allocation/ 16. exp Single-Blind Method/ 17. exp Double-Blind Method/ 18. 12 or 13 or 14 or 15 or 16 or 17 19. 4 and 11 and 18 |
| --- |

# Figure S1. PRISMA flow diagram

^
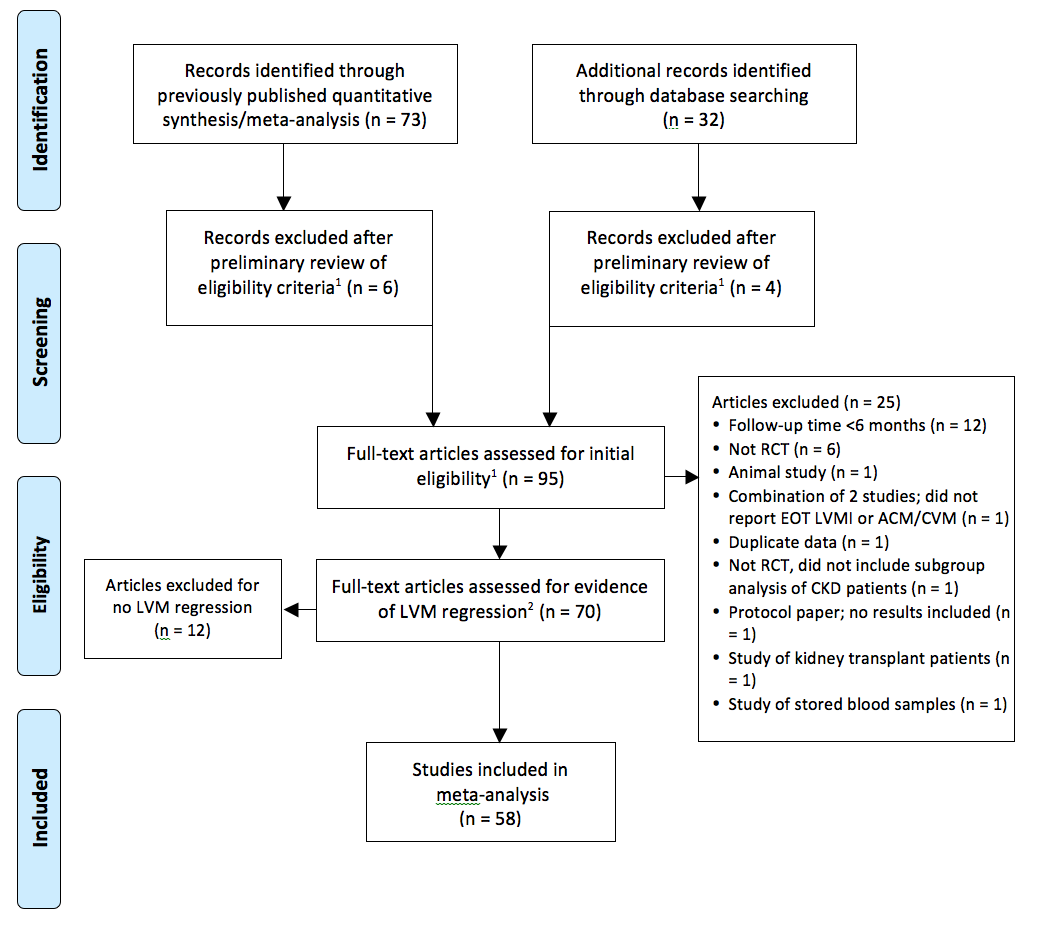
^

^1^Preliminary and initial eligibility assessment included: inclusion criteria: RCTs that reported treatment effects on LVM in adults or children with any stage of CKD and randomized trials that reported treatment effects on LVM in adults or children in the general population that included a separate subgroup analysis of participants with CKD; exclusion criteria: observational studies; trials with follow-up duration <6 months; or trials involving kidney transplant recipients.

^2^LVM regression was defined as a mean change in standardized LVM (usually expressed as LVMI) ≤-0.01 standard deviations for the intervention group change minus the control group change.

Abbreviations: ACM = all-cause mortality; CKD = chronic kidney disease; CVM = cardiovascular mortality; EOT = end of treatment; LVM = left ventricular mass; LVMI = left ventricular mass index; RCT = randomized controlled trial.

# Table S2. Individual study details

| **Author, Year** | **ACM & CVM reported (Y/N)** | **Intervention** | **Sub-groups** | **Total RND (n)** | **Tx RND (n)** | **Control RND (n)** | **F/U (months)** | **Inclusion Criteria** | **Intervention details** | **Control details** | **Method to measure LVM** |
| --- | --- | --- | --- | --- | --- | --- | --- | --- | --- | --- | --- |
| Levin 2005 | ACM-Y CVM-N | ESA Higher Hb |  | 172 | 85 | 87 | 24 | - Age 18-75 yrs - CrCl 15-79 mL/min - Decline in Hb ≥1 g/dL within 12 months to current Hb 11-13.5 g/dL for men and 11.5-12.5 g/dL for women, OR current Hb 11.5-12.5 g/dL for men and 11- 12 g/dL for women - *eGFR 29 mL/min/1.73 m^2^* | Target Hb  12-14 g/dL | Target Hb  9-10.5 g/dL | Echo |
| Parfrey 2005 | ACM-Y CVM-N | ESA Higher Hb |  | 596 | 296 | 300 | 24 | - Age ≥18 yrs, - ESKD on HD for 3-18 months - Hb 8-12 g/dL - LVVI<100 mL/m^2^ - diastolic BP<100 mm Hg | Target Hb  13.5-14.5 g/dL | Target Hb  9.5-11.5 g/dL | Echo |
| MacDougall 2007 | ACM- Y CVM-N | ESA Higher Hb |  | 197 | 65 | 132 | 36 | - Age 18-85 yrs, - Progressive increase in serum creatinine from 150-500 µmol/L over ≥3 months before enrollment - progressive decrease in Hb from 11±0.5 g/dL over ≥3 months before enrollment - *eGFR 20 mL/min/1.73 m^2^* | Early EPO start Target Hb  11±1 g/dL | Late EPO start  Target Hb  11±1 g/dL | Echo |
| Ritz 2007 | Both-N | ESA Higher Hb |  | 172 | 89 | 83 | 15 | - Age ≥18 yrs - CrCl ≥30 mL/min - Type 1 or 2 DM - Documented diabetic nephropathy - Hb 10.5-13 g/dL - BP≤140/90 mm Hg - *CrCl 49 mL/min* | Target Hb  13-15 g/dL | Target Hb  10.5-11.5 g/dL | Echo |
| Pappas 2008 | Both-Y | ESA Higher Hb |  | 31 | 15 | 16 | 12 | - Age >18 yrs - CrCl 15-59 mL/min - Hb<12 g/dL - *CrCl 25 mL/min* | Target Hb  >13 g/dL | Target Hb  >9 g/dL | Echo |
| Cianciaruso 2008 | Both-Y | ESA Higher Hb |  | 95 | 46 | 49 | 24 | - Age 18-75 yrs - CrCl 15-79 mL/min - Decline in Hb ≥1 g/dL within 12 months to current Hb 11-13.5 g/dL for men and 11.5-12.5 g/dL for women, or current Hb 11.5- 12.5 g/dL for men and 11-12 g/dL for women - *eGFR 26 mL/min/1.73 m^2^* | Target Hb  12-14 g/dL | Target Hb  9-10.5 g/dL | Echo |
| Akizawa 2011 | ACM- Y CVM-N | ESA Higher Hb |  | 322 | 161 | 161 | 8 | - Age ≥20 yrs - Serum creatinine 2.0 to 6.0 mg/dL - Hb <10 g/dL - *eGFR 12 mL/min/1.73 m^2^* | Target Hb  11-13 g/dL | Target Hb  9-11 g/dL | Echo |
| Suzuki 2002 | No deaths | RAASi vs. placebo or  stnd tx | Without LVH | 24 | 12 | 12 | 12 | - Type 2 DM - HTN - CrCl 14-35 mL/min - Data on BL kidney function not reported | Benazepril  5 mg daily | No benazepril | Echo |
|  |  |  |  | 24 | 12 | 12 | 12 |  | Benazepril  2.5 mg daily | No benazepril |  |
|  |  |  | With LVH | 24 | 12 | 12 | 12 |  | Benazepril  5 mg daily | No benazepril |  |
|  |  |  |  | 24 | 12 | 12 | 12 |  | Benazepril  2.5 mg daily | No benazepril |  |
| Suzuki 2003 | No deaths | RAASi vs. placebo or  stnd tx |  | 24 | 14 | 10 | 12 |  |  |  | Echo |
| Kanno 2004 | No deaths | RAASi vs. placebo or  stnd tx |  | 24 | 12 | 12 | 12 | - Type 2 DM - ESKD due to diabetic nephropathy - Newly starting HD - LVH | Losartan | Placebo | Echo |
| Matsumoto 2006 | No deaths | RAASi vs. placebo or  stnd tx |  | 30 | 15 | 15 | 6 | - ESKD on HD | Imidapril | Placebo | Echo |
| Yu 2006 | Both-Y | RAASi vs. placebo or  stnd tx |  | 46 | 24 | 22 | 12 | - ESKD on HD for ≥6 months - BP <140/90 mm Hg without antihypertensive drugs | Ramipril | Placebo | Echo |
| Mitsuhashi 2009 | No deaths | RAASi vs. placebo or  stnd tx |  | 40 | 20 | 20 | 12 | - ESKD on HD for >2 wks - post-HD BP > 140/90 mm Hg | Losartan | Stnd tx | Echo |
| Taheri 2009 | Both-Y | RAASi vs. placebo or  stnd tx |  | 16 | 8 | 8 | 6 | - ESKD on HD | Spironolactone | Placebo | Echo |
| Ito 2014 | Both-Y | RAASi vs. placebo or  stnd tx |  | 158 | 78 | 80 | 24 | - Age 18-80 yrs - ESKD on PD with neutral pH dialysate AND/OR - Icodextrin - Tx with ACE inhibitor or ARB for > 3 months | Initially spironolactone,  later changed to eplerenone | Stnd tx | Echo |
| Feniman-  De-Stefano 2015 | No deaths | RAASi vs. placebo or  stnd tx |  | 19 | 10 | 9 | 6 | - Age ≥18 yrs - ESKD on HD - LVMI ≥51 g/m^2^ | Spironolactone | Placebo | Echo |
| London 1994 | No deaths | RAASi vs. placebo or  stnd tx |  | 32 | 16 | 16 | 12 | - ESKD on HD for >6 months - BP >160/95 mm Hg - LVH (LVMI ≥134 g/m^2^-men and ≥110 g/m^2^-women) | Perindopril | Nitrendipine | Echo |
| Shibasaki 2002 | No deaths | RAASi vs. placebo or  stnd tx |  | 20 | 10 | 10 | 6 | - ESKD for HD for ≥1 month - BP >150/90 mm Hg | Losartan | Amlodipine | Echo |
| Zeltner 2008 | No deaths | RAASi vs. placebo or  stnd tx |  | 46 | 23 | 23 | 36 | - Age 18-65 yrs - ADPKD - BP ≥140/90 mm Hg or antihypertensive drugs - Serum creatinine ≤4 mg/dL - *eGFR 88 mL/min/1.73 m^2^* | Ramipril | Metoprolol | Echo |
| Yilmaz 2010 | ACM- Y CVM-N | RAASi vs. placebo or  stnd tx |  | 112 | 56 | 56 | 12 | - ESKD on HD - BP >140/90 mm Hg AND/OR - Use of antihypertensive drugs | Ramipril | Amlodipine | Echo |
| Ulusoy 2010 | No deaths | RAASi vs. other RAASi |  | 32 | 19 | 13 | 12 | - Age 18-70 yrs - ADPKD - CrCl ≥30 mL/min/1.73 m^2^ - BP ≥140/90 mm Hg or on antihypertensive drugs - *CrCl 78 mL/min/1.73 m^2^* | Ramipril | Losartan | Echo |
| Schrander-vd Meer 1999 | No deaths | Convective HD |  | 24 | 12 | 12 | 12 | - ESKD stable on bicarbonate HD for ≥1 yr | Acetate-free biofiltration (Postdilution HDF) | High-flux HD | Echo |
| Alvestrand 2011 | ACM- Y CVM-N | Convective HD |  | 48 | 27 | 21 | 24 | - Age 18-80 yrs - ESKD on HD for <3 months | Online predilution hemofiltration | Low-flux HD | Echo |
| Ohtake 2012 | No deaths | Convective HD |  | 22 | 13 | 9 | 12 | - ESKD on HD for >6 months | Online HDF | High-flux HD | Echo |
| Maduell 2012 | No deaths | Convective HD |  | 26 | 14 | 12 | 6 | - ESKD on HD - Good vascular access | High convective volume HDF | Standard convective volume HDF | Echo |
| Mostovaya 2014 | Both-Y | Convective HD |  | 714 | 358 | 356 | 12 | - Age ≥18 yrs - ESKD on HD for ≥2 months | Online post-dilation HDF | Low-flux HD | Echo |
|  |  |  |  | 331 | 164 | 167 | 48 |  |  |  |  |
| Culleton 2007 | ACM- Y CVM-N | ≥4x/wk HD |  | 52 | 27 | 25 | 6 | - Age >18 yrs, - ESKD on HD - Willing to train and commence nocturnal HD | HD 5-6 times/wk  (>6 hrs/session) | HD 3 times/wk | cMRI |
| Katopodis 2009 | No deaths | ≥4x/wk HD |  | 18 | 9 | 9 | 12 | - ESKD on HD for >6 months | HD every other day | HD 3 times/wk | Echo |
| Chertow 2010 | Both-Y | ≥4x/wk HD |  | 245 | 125 | 120 | 12 | - Age ≥13 yrs - ESKD - Weight ≥30 kg - Adequate dialysis dose | HD 6 times/wk  (1.5-2.75 hrs/session) | HD 3 times/wk  (2.5-4 hrs/ session) | cMRI |
| Rocco 2011 | Both-Y | ≥4x/wk HD |  | 87 | 45 | 42 | 12 | - Age ≥18 yrs - ESKD requiring renal replacement therapy - Willing to perform HD at home | Frequent nocturnal HD 6 times/wk  (>6 hrs/session) | HD 3 times/wk | MRI |
| Ivarsen 2012 | No deaths | Vit D compound |  | 14 | 7 | 7 | 6 | - CKD stage 4 - PTH >3 times upper normal level - Serum phosphate <2 mmol/L - Serum calcium <1.35 mmol/L - LVH - BP <160/95 mm Hg - *CrCl 23 mL/min* | Alfacalcidol | No tx | Echo |
| Dreyer 2014 | No deaths | Vit D compound |  | 38 | 20 | 18 | 6 | - Age 18-80 yrs - eGFR 15-60 mL/min/1.73 m^2^ - 25 (OH) vitamin D level <16 ng/mL (<40 nmol/L) - *eGFR 36 mL/min/1.73 m^2^* | Ergocalciferol | Placebo | cMRI |
| Howden 2013 | No deaths | Exercise |  | 83 | 41 | 42 | 12 | - Age 18-75 yrs - eGFR 25-60 mL/min/1.73 m^2^ - Uncontrolled HTN or BMI ≥25 kg/m^2^ or HbA1c >7% or lipids exceeding target - *eGFR 39 mL/min/1.73 m^2^* | Exercise training and lifestyle intervention | Usual lifestyle | Echo |
| Liu 2014 | No deaths | Fluid mgmt |  | 56 | 28 | 28 | 6 | - ESKD newly starting HD - Urine output >1 L/day | Ultrafiltration during HD | No ultrafiltration during HD | Echo |
| Hur 2013 | ACM- Y CVM-N | Fluid mgmt |  | 156 | 78 | 78 | 12 | - Age >18 yrs - ESKD on HD for > 3 months | Fluid management based on bioimpedance spectroscopy | Fluid management based on routine care | Echo |
| Schrier 2002 | ACM- Y CVM-N | Lower BP |  | 79 | 42 | 37 | 84 | - Age 20-60 yrs - ADPKD - CrCl ≥30mL/min/1.73 m^2^ - BP ≥140/90 mm Hg - LVH (LVMI ≥125 g/m^2^-men and ≥110 g/m^2^-women) - CrCl 83 mL/min/1.73 m^2^ | Target BP <120/80 mm Hg | Target BP  135-140/  85-90 mm Hg | Echo |
| Li 2011 | No deaths | Inosorbide monoitrate |  | 144 | 72 | 72 | 6 |  | Inosorbide monoitrate |  | Echo |
| Li 2013 | No deaths | Inosorbide monoitrate |  | 64 | 32 | 32 | 6 |  | Inosorbide monoitrate |  | Echo |
| Feldt-Rasmussen 2007 | ACM- Y CVM-N | Growth Hormone |  | 68 | 34 | 34 | 6 |  | 20 ug/kg/d | Placebo | Echo |
|  |  |  |  | 68 | 34 | 34 | 6 |  | 35 ug/kg/d | Placebo |  |
|  |  |  |  | 71 | 37 | 34 | 6 |  | 50 ug/kg/d | Placebo |  |
| Nakamura 2002 | No deaths | Misc  Dilazep |  | 40 | 20 | 20 | 12 | - ESKD on HD - LVH (LVMI >125 g/m2) | Dilazep dihydrochloride | Placebo | Echo |
| Hotu 2010 | Both-Y | Misc |  | 65 | 33 | 32 | 12 | - Maori or Pacific ethnicity - Type 2 DM - Age 40-75 yrs - Proteinuria >0.5 g/day - Serum creatinine 130-300 µmol/L - BP >130/80 mm Hg - *eGFR 37 mL/min/1.73 m^2^* | Nurse-led, community- based, integrated care | Physician-led, clinic-based care | Echo |
| Chen 2011 |  | Misc |  | 100 | 51 | 49 | 24 | - ESKD on HD for 6-30 months - Urine output <500 mL/day | HD twice/wk and HD plus hemoperfusion once/wk | HD 3 times/wk | ultrasonic cardiogram |
| Kao 2011 | No deaths | Misc |  | 67 | 32 | 35 | 9 | - CKD stage 3 - LVH (LVMI ≥115 g/m^2^ in men and ≥95 g/m^2^ in women) - *eGFR 45 mL/min/1.73 m^2^* | Allopurinol | Placebo | MRI |
| Zamboli 2011 | No deaths | Misc |  | 40 | 20 | 20 | 12 | - Age ≥18 yrs - CrCl 15-60 mL/min/1.73 m^2^ - Tx with ACE inhibitor or ARB - Systolic BP >140 mm Hg - *eGFR 38 mL/min/1.73 m^2^* | Furosemide | No tx | Echo |
| Whalley 2013 | ACM- Y CVM-N | Misc |  | 182 | 91 | 91 | 12 | - Age ≥18 yrs - Progressive renal failure - GFR 10-15 mL/min/1.73 m^2^ - *eGFR 9.7 mL/min/1.73 m^2^* | Early dialysis start (GFR 10-14 mL/min/1.73m^2^) | Late dialysis start (GFR 5-7 mL/min/1.73 m^2^) | Echo |
| Odudu 2015 | ACM- Y CVM-N | Misc |  | 73 | 36 | 37 | 12 | - Age ≥16 yrs - ESKD within 180 days of commencing in-center HD | Cooling of dialysate temp. to 0.5^○^C below body temp. | Dialysate temperature 37^○^C | cMRI |
| Higuchi 2016 | ACM- Y CVM-N | Misc levocarnitine |  | 222 | 110 | 112 | 12 | - Age 20 to 85 yrs - ESKD on HD for > 6 months - Free carnitine plasma concentration <40 umol/L | Levocarnitine | No tx | Echo |
| Jardine 2017 | ACM-Y CVM-N | HD |  | 200 | 100 | 100 | 12 | - Incident or prevalent dialysis patients not currently receiving extended hrs (>18 hrs per wk) - Life expectancy ≥ 6 months - No planned renal transplantation within 12 months | Extended HD  24 hrs/wk | Standard HD  12 hrs/wk | MRI |
| Liu 2016 | ACM-Y CVM-N | Misc  Reduced dialysate sodium |  | 64 | 32 | 32 | 12 | - HD >6 months - LVEF >40% - Serum albumin 3g/dL - Hypertensive - Interdialytic BP 135/85 mm Hg - No change to antihypertensive tx in month before enrollment | 136mmol/L  dialysate sodium | 138 mmol/L dialysate sodium | Echo |
| Lin 2016 | ACM-Y CVM-Y | Misc Spironolactone | Men | 116 | 73 | 80 | 24 | - ESKD on HD 3 times/wk (4-4.5 hrs/session) OR maintenance PD and CAPD - Age>18yrs | Spironolactone  25 mg, once/day | Placebo | Echo |
|  |  |  | Women | 82 | 52 | 48 | 24 |  |  |  |  |
| Charytan 2019 | ACM-Y  CVM-Y | Misc  Spironolactone |  | 129 | 27 | 51 | 36 | - 18-85 yrs - Maintenance HD ≥6 mos or 3-6 mos if no changes in target dry weight during prior 2 wks and no hospitalizations during prior 6 wks | 12.5 mg | Placebo | Echo |
|  |  |  |  |  | 26 | 51 |  |  | 25 mg |  |  |
|  |  |  |  |  | 25 | 51 |  |  | 50 mg |  |  |
| Miskulin 2018 | ACM-Y  CVM-Y | Lower BP |  | 126 | 62 | 64 | 12 | - ≥ 18 yrs - On HD for ≥ 3 months - Upper arm suitable for measuring BP - 2-wk average predialysis SBP ≥ 155 mm Hg | Treatment to standardized predialysis SBP of 110-140 mm Hg | Treatment to standardized predialaysis SBP of 155-165 mm Hg | Cardiac MRI |
| Djuric 2020 | ACM-Y  CVM-Y | Misc  Sodium thiosulphate |  | 60 | 30 | 30 | 6 | - > 18 yrs - On chronic thrice-weekly HD for > 6 months - Abdominal aortic calcification score ≥ 100 Agaston units | Sodium thiosulphate 25 g/1.73 m2 during last 15 min of every HD session | 100 mL of 0.9% NaCl IV during last 15 min of every HD session | Echo |
| Marshall 2020 | ACM-Y  CVM-N | HD  Low-Na vs. conventional Na |  | 99 | 49 | 50 | 12 | - > 18 yrs - Pre-dialysis serum sodium ≥135 mM - Receiving HD at home or self-care satellite facility | 135 mmol/L dialysate sodium | 140 mmol/L dialysate sodium | Cardiac MRI |
| Fujii 2018 | ACM-Y  CVM-Y |  |  | 105 | 50 | 55 | 12, 18 | - ≥ 20 yrs - Presence of hyperphosphatemia | Lanthanum carbonate | Calcium carbonate | Echo |
| Rutherford 2021 | ACM-Y  CVM-N |  |  | 80 | 40 | 40 | 12 | - > 18 yrs - On HD > 3 months | Allopurinol 100 mg thrice weekly for 2 weeks; if tolerated, escalated weekly to 200 mg, 250 mg, up to max 300 mg | Placebo capsule thrice weekly | Cardiac MRI |
| Dorr 2021 | ACM-Y  CVM-N |  |  | 62 | 32 | 30 | 12 | - Initiated dialysis in the last 3 to 36 months - had LVH, defined as a septum thickness of a minimum of 12 mm in echocardiography - sHPT, defined as PTH ≥300 ng/L - Stable volume status (i.e., achieved and tolerated individual optimal dry weight at the end of dialysis) | Etelcalcetide thrice/week | Alfacalcidol thrice/week | Cardiac MRI |
| Edwards 2021 | ACM-Y  CVM-N |  |  | 154 | 77 | 77 | 12 | - ≥ 18 yrs - Stable CKD stage 2 or 3 (eGFR 30-89ml/min/1.73m2) - Taking an ACE inhibitor or ARB with controlled BP using standard UK guideline blood pressure target values <130/80mmHg in 2015 | Spironolactone 25mg once daily | Chlorthalidone 25mg once daily | Cardiac MRI |

| **Author, Year**  **(cont)** | **Sub-groups** | **Mean BL LVM/ LVMI*** | **Difference in ∆ from BL*** | **SD at BL, pooled** | **SMD*** | **Tx events (ACM)** | **Control events (ACM)** | **Tx mean/range eGFR at BL** | **Control mean/range eGFR at BL** | **eGFR units** | **Notes** |
| --- | --- | --- | --- | --- | --- | --- | --- | --- | --- | --- | --- |
| Levin 2005 |  | 99.45 | -4.84 | 23.64 | -0.20 | 1 | 3 | 29.70 | 27.80 | mL/min | Mean LVM ∆ from BL reported in the original paper |
| Parfrey 2005 |  | 114.25 | -4.20 | 34.37 | -0.12 | 13 | 20 | <15 | | mL/min | Tx is higher Hb group, Control is lower Hb group; LVM ∆ from BL = mean LVM at EOS minus mean LVM at BL |
| MacDougall 2007 |  | 259.75 | -23.20 | 92.42 | -0.25 | 1 | 6 | 25.75 | 23.26 | mL/min | LVM ∆ from BL = mean LVM at EOS minus mean LVM at BL |
| Ritz 2007 |  | 114.75 | -1.70 | 32.66 | -0.05 | 0 | 0 | ≥30 | | mL/min | LVMI ∆ from BL = mean LVMI at EOS minus mean LVMI at BL; BL SDs reported in original paper; inclusion criteria= CKD stages 1-3 |
| Pappas 2008 |  | 137.80 | -51.10 | 52.55 | -0.97 | 1 | 3 | 15-59 | | mL/min | LVM ∆ from BL = mean LVM at EOS minus mean LVM at BL; inclusion criteria= CKD stages 3-4 |
| Cianciaruso 2008 |  | 109.10 | -1.93 | 26.17 | -0.07 | 1 | 0 | 26.20 | 26.70 | mL/min | Mean LVM ∆ from BL reported in the original paper |
| Akizawa 2011 |  | 127.00 | -7.70 | 36.17 | -0.21 | 1 | 0 | 12.55 | 12.34 | mL/min/ 1.73 m^2^ | 161 randomized to control (low Hb group), but one subject withdrew consent prior to tx; Mean LVM ∆ from BL reported in the original paper |
| Suzuki 2002 | Without LVH, 5 mg benazepril | 96.50 | -19.00 | 36.74 | -0.52 | 0 | 0 | NA | NA |  | Control is Group III comprised patients not taking benazepril; LVMI ∆ = mean LVMI at EOS minus mean LVMI at BL; BL SEMs reported in original paper, SDs calculated |
|  | Without LVH, 2.5 mg benazepril | 97.00 | -17.00 | 39.87 | -0.43 | 0 | 0 | NA | NA |  |  |
|  | With LVH, 5 mg benazepril | 116.50 | -16.00 | 36.74 | -0.44 | 0 | 0 | NA | NA |  | Control is Group III-L comprised patients not taking benazepril; LVMI ∆ = mean LVMI at EOS minus mean LVMI at BL; BL SEMs reported in original paper, SDs calculated |
|  | With LVH, 2.5 mg benazepril | 117.00 | -6.00 | 39.87 | -0.15 | 0 | 0 | NA | NA |  |  |
| Suzuki 2003 |  | 148.50 | -9.00 | 14.83 | -0.61 | 0 | 0 | <15 | | mL/min | LVMI ∆ from BL = mean LVMI at EOS minus mean LVMI at BL; variability stats reported in paper assumed to be SEMs; subjects had ESKD= CKD stage 5 |
| Kanno 2004 |  | 146.00 | -15.00 | 14.28 | -1.05 | 0 | 0 | <15 | | mL/min | LVMI ∆ from BL = mean LVMI at EOS minus mean LVMI at BL; SEM of BL LVMI reported in paper, SD calculated; subjects had ESKD= CKD stage 5 |
| Matsumoto 2006 |  | 130.50 | -20.00 | 31.94 | -0.63 | 0 | 0 | <15 | | mL/min | LVMI ∆ from BL = mean LVMI at EOS minus mean LVMI at BL; SEM of BL LVMI reported in paper, SD calculated; subjects had ESKD= CKD stage 5 |
| Yu 2006 |  | 108.50 | -12.00 | 23.63 | -0.51 | 1 | 0 | <15 | | mL/min | LVM ∆ from BL = mean LVM at EOS minus mean LVM at BL; Values for EOS were 12-month values (not washout); subjects had ESKD= CKD stage 5 |
| Mitsuhashi 2009 |  | 183.00 | -42.00 | 47.01 | -0.89 | 0 | 0 | <15 | | mL/min | LVM ∆ from BL = mean LVM at EOS minus mean LVM at BL; SEM of BL LVM reported in paper, SD calculated |
| Taheri 2009 |  | 158.69 | -11.40 | 14.51 | -0.79 | 3 | 2 | <15 | | mL/min | LVM ∆ from BL: Mean ∆ from BL reported in the original paper; variability stat assumed to be SDs |
| Ito 2014 |  | 51.95 | -6.78 | 19.65 | -0.34 | 2 | 5 | <15 | | mL/min | % ∆ in LVMI reported in a graph, neither EOS nor ∆ from BL values reported in the text or in a table; Abs ∆ from BL = % ∆ (pulled from graph using Engague Digitizer software) x BL value/100 |
| Feniman-De-Stefano 2015 |  | 74.00 | -11.00 | 14.40 | -0.76 | 0 | 0 | <15 | | mL/min | LVMI ∆ from BL = mean LVMI at EOS minus mean LVMI at BL |
| London 1994 |  | 185.00 | -29.00 | 50.99 | -0.57 | 0 | 0 | <15 | | mL/min | LVMI ∆ from BL = mean LVMI at EOS minus mean LVMI at BL; SEM of BL LVMI reported in paper, SD calculated; subjects had ESKD = CKD stage 5 |
| Shibasaki 2002 |  | 155.55 | -14.20 | 27.50 | -0.52 | 0 | 0 | <15 | | mL/min | Mean LVMI ∆ from BL reported in the original paper; SEM of BL LVMI reported in paper, SD calculated; subjects had ESKD = CKD stage 5 |
| Zeltner 2008 |  | 96.30 | -0.30 | 25.12 | -0.01 | 0 | 0 | 88.00 | 87.30 | mL/min | LVMI ∆ from BL = mean LVMI at EOS minus mean LVMI at BL; SEM of BL LVMI reported in paper, SD calculated |
| Yilmaz 2010 |  | 125.00 | -7.00 | 39.00 | -0.18 | 0 | 1 | <15 | | mL/min | LVMI ∆ from BL = mean LVMI at EOS minus mean LVMI at BL; subjects had ESKD = CKD stage 5 |
| Ulusoy 2010 |  | 119.00 | -8.59 | 17.59 | -0.49 | 0 | 0 | NA | NA |  | LVMI ∆ from BL = mean LVMI at EOS minus mean LVMI at BL; BL SDs reported in original paper |
| Schrander-vd Meer 1999 |  | 174.60 | -62.00 | 89.25 | -0.69 | 0 | 0 | <15 | | mL/min | LVMI ∆ from BL = mean LVMI at EOS minus mean LVMI at BL; BL SDs reported in original paper |
| Alvestrand 2011 |  | 156.00 | -16.00 | 51.16 | -0.31 | 2 | 3 | <15 | | mL/min | LVMI ∆ from BL = mean LVMI at EOS minus mean LVMI at BL; inclusion criteria were CKD stage 5 |
| Ohtake 2012 |  | 139.95 | -9.70 | 37.97 | -0.26 | 0 | 0 | <15 | | mL/min | LVMI ∆ from BL = mean LVMI at EOS minus mean LVMI at BL; subjects had ESKD= CKD stage 5 |
| Maduell 2012 |  | 139.00 | -25.00 | 34.53 | -0.72 | 0 | 0 | <15 | | mL/min | Cross-over study, only tx period 1 used in meta-analysis; LVMI ∆ from BL = mean LVMI at EOS minus mean LVMI at BL; subjects with ESKD = CKD stage 5 |
| Mostovaya 2014 | 24 month F/U | 125.00 | -4.45 | 38.77 | -0.11 | 41 | 51 | <15 | | mL/min | LVMI ∆ from BL in original paper; SD values not provided, only 25th and 75th percentiles, SD was estimated assuming distribution is normal as SD=Abs[(75th percentile-25th percentile)/(Z(.75)-Z(.25))]; subjects with ESKD = CKD stage 5 |
|  | 48 month F/U | 125.00 | -27.72 | 38.77 | -0.71 | 41 | 51 | <15 | | mL/min |  |
| Culleton 2007 |  | 97.10 | -8.10 | 40.42 | -0.20 | 1 | 0 | <15 | | mL/min | Mean LVMI ∆ from BL reported in original paper; BL SDs reported in original paper |
| Katopodis 2009 |  | 268.35 | -36.50 | 144.19 | -0.25 | 0 | 0 | <15 | | mL/min | LVMI ∆ from BL = mean LVM at EOS minus mean LVM at BL; BL SDs reported in original paper |
| Chertow 2010 |  | 141.50 | -13.90 | 54.23 | -0.26 | 5 | 9 | <15 | | mL/min | Mean LVMI ∆ from BL reported in original paper; Unadjusted mean ∆ from BL was used in SMD calculation; BL SDs reported in original paper |
| Rocco 2011 |  | 136.50 | -8.80 | 44.64 | -0.20 | 2 | 1 | <15 | | mL/min | Mean LVMI ∆ from BL reported in original paper; Unadjusted mean ∆ from BL was used in SMD calculation, BL SDs reported in original paper |
| Ivarsen 2012 |  | 141.45 | -1.70 | 26.88 | -0.06 | 0 | 0 | 15-29 | | mL/min | LVM ∆ from BL = mean LVM at EOS minus mean LVM at BL; BL SEMs reported in original paper, SDs calculated; subjects with CKD stage 4 |
| Dreyer 2014 |  | 91.80 | -23.90 | 28.46 | -0.84 | 0 | 0 | 33.00 | 38.70 | ml/min/ 1.73 m^2^ | LVM ∆ from BL = mean LVM at EOS minus mean LVM at BL; BL SDs reported in original publication; BL SD for control group reported as 174 in original paper, and assumed to be an error, SMD calculation used 17.4 as the BL SD for the control group |
| Howden 2013 |  | 54.90 | -0.90 | 14.42 | -0.06 | 0 | 0 | 38.40 | 39.40 | ml/min/ 1.73 m^2^ | Mean LVMI ∆ from BL reported in original paper; BL SDs reported in original paper |
| Liu 2014 |  | 125.05 | -21.60 | 19.35 | -1.12 | 0 | 0 | <15 | | mL/min | Mean LVMI ∆ from BL reported in original paper; BL SDs reported in original paper |
| Hur 2013 |  | 126.00 | -14.00 | 35.50 | -0.39 | 2 | 4 | <15 | | mL/min | LVMI ∆ from BL = mean LVMI at EOS minus mean LVMI at BL; BL SDs provided in original paper |
| Schrier 2002 |  | 158.50 | -23.59 | 25.54 | -0.92 | 1 | 1 | NA | NA |  | LMVI decreased by 21% in control and 35% in intervention, ∆ from BL = -(LVMI at BL) x (decrease as decimal); BL SDs reported in original paper |
| Li 2011 |  | 64.50 | -21.60 | 15.50 | -1.39 | 0 | 0 | <15 | | mL/min | Mean LVMI ∆ from BL reported in original paper; BL SDs reported in original paper; subjects with ESKD = CKD stage 5 |
| Li 2013 |  | 64.65 | -4.00 | 14.25 | -0.28 | 0 | 0 | <15 | | mL/min | Mean LVMI ∆ from BL reported in original paper; BL SDs reported in original paper; subjects with ESKD = CKD stage 5 |
| Feldt-Rasmussen 2007 | hGH 20 ug/kg/d | 205.00 | -6.60 | 73.98 | -0.09 | 2 | 4 | <15 | | mL/min | Control is placebo; mean ∆s from BL reported in original paper; BL SDs reported in original paper; subjects had ESKD = CKD stage 5 |
|  | hGH 35 ug/kg/d | 183.00 | -9.50 | 60.50 | -0.16 | 3 | 4 | <15 | | mL/min |  |
|  | hGH 50 ug/kg/d | 203.00 | -5.10 | 65.12 | -0.08 | 3 | 4 | <15 | | mL/min |  |
| Nakamura 2002 |  | 176.50 | -11.00 | 47.01 | -0.23 | 0 | 0 | <15 | | mL/min | LVMI ∆ from BL = mean LVMI at EOS minus mean LVMI at BL; BL SDs provided in original paper |
| Hotu 2010 |  | 140.65 | -20.20 | 29.44 | -0.69 | 2 | 0 | 36.00 | 39.00 | ml/min/ 1.73 m^2^ | LVMI ∆ from BL = mean LVMI at EOS minus mean LVMI at BL; BL SDs provided in original paper |
| Chen 2011 |  | 104.49 | -71.23 | 12.95 | -5.50 | 6 | 14 | <15 | | mL/min | LVMI ∆ from BL = mean LVMI at EOS minus mean LVMI at BL; BL SDs provided in original paper |
| Kao 2011 |  | 61.85 | -2.70 | 14.57 | -0.19 | 0 | 1 | 44.00 | 46.00 | ml/min/ 1.73 m^2^ | Mean LVMI ∆ from BL reported in original paper; BL SDs reported in original paper |
| Zamboli 2011 |  | 62.45 | -9.70 | 17.77 | -0.55 | 0 | 0 | 38.90 | 35.70 | ml/min/ 1.73 m^2^ | LVMI ∆ from BL = mean LVMI at EOS minus mean LVMI at BL; BL SDs provided in original paper |
| Whalley 2013 |  | 135.40 | -7.80 | 39.55 | -0.20 | 2 | 1 | 13.00 | 13.00 | ml/min/ 1.73 m^2^ | LVMI ∆ from BL = mean LVMI at EOS minus mean LVMI at BL; BL SDs provided in original paper; subjects CKD stage 5 |
| Odudu 2015 |  | 76.55 | -6.80 | 19.85 | -0.34 | 2 | 1 | <15 | | mL/min | LVMI ∆ from BL = mean LVMI at EOS minus mean LVMI at BL; BL SDs provided in original paper |
| Higuchi 2016 |  | 111.00 | -10.00 | 25.02 | -0.40 | 5 | 7 | <15 | | mL/min | LVMI ∆ from BL = mean LVMI at EOS minus mean LVMI at BL; BL SDs provided in original paper |
| Jardine 2017 |  | 105.05 | -6.50 | 33.67 | -0.19 | 5 | 2 | <15 | | mL/min | LVMI ∆ from BL = mean LVMI at EOS minus mean LVMI at BL; BL SDs provided in original paper |
| Liu 2016 |  | 150.00 | -10.00 | 18.03 | -0.55 | 3 | 2 | <15 | | mL/min | LVMI ∆ from BL = mean LVMI at EOS minus mean LVMI at BL; BL SDs provided in original paper |
| Charytan 2019 | Spironolactone 12.5 mg | 110.35 | -0.5 | 25.96 | -0.02 | 0 | 2 | NA | |  | Mean changes from BL for each condition were reported in the original paper  BL SDs for each condition were reported in the original paper |
|  | Spironolactone 25 mg | 110.80 | 3.1 | 26.06 | 0.12 | 2 |  |  |  |  |  |
|  | Spironolactone 50 mg | 105.75 | 0.6 | 27.27 | 0.02 | 1 |  |  |  |  |  |
| Lin 2016 | Men | 52.59 | -10.10 | 19.60 | -0.52 | unknown | unknown | <15 | | mL/min | 58.4% of intervention group were male; 62.5% of control group were male; # females in each group = total # minus # males; LVMI ∆ from BL = mean LVMI at EOS minus mean LVMI at BL; BL SDs provided in original paper |
|  | Women | 47.33 | -8.71 | 14.90 | -0.58 | unknown | unknown | <15 | | mL/min |  |
|  | Total |  |  |  | -0.55 | 12 | 25 | <15 | | mL/min |  |
| Miskulin 2018 |  | 146.65 | -2.24 | 46.80 | -0.05 | 4 | 1 | NA | |  | Median and 25^th^ and 75^th^ percentiles for LVMI ∆ were reported in original paper. Mean LVMI ∆ estimated as median LVMI ∆. SD for LVMI ∆ was estimated assuming distribution is normal as SD = Abs[(75th percentile-25th percentile)/(Z(.75)-Z(.25))]. |
| Djuric 2020 |  | 11.05 | -0.6 | 1.92 | -0.31 | 1 | 0 | NA | |  | LVMI ∆ from BL = mean LVMI at EOS minus mean LVMI at BL; BL SDs provided in original paper |
| Marshall 2020 |  | 94.00 | -3.94 | 26.23 | -0.15 | 2 | 1 | NA | |  | Difference in ∆ from BL was reported in original paper  BL SDs were reported in original paper |
| Fujii 2018 | 12 month F/U | 157.30 | -0.70 | 41.00 | -0.02 | 1 | 1 | 52 | 57 | ml/min/ 1.73 m^2^ | LVMI ∆ from BL = mean LVMI at EOS minus mean LVMI at BL; BL SDs provided in original paper |
|  | 18 month F/U | 157.30 | -2.70 | 41.00 | -0.07 | 1 | 1 | 52 | 57 | ml/min/ 1.73 m^2^ | LVMI ∆ from BL = mean LVMI at EOS minus mean LVMI at BL  BL SDs provided in original paper |
| Rutherford 2021 |  | 60.75 | -2.10 | 21.75 | -0.10 | 2 | 5 | NA | |  | Mean LVMI ∆ from BL and mean difference in LVMI ∆ reported in original paper  Median and 25^th^ and 75^th^ percentiles for BL were reported in original paper. Mean BL estimated as median BL. SD for BL estimated assuming distribution is normal as SD = Abs[(75th percentile-25th percentile)/(Z(.75)-Z(.25))]. |
| Dorr 2021 |  | 69.50 | -6.90 | 17.10 | -0.40 | 2 | 1 | NA | |  | Mean LVMI ∆ from BL and mean difference in LVMI ∆ reported in original paper  Median and 25^th^ and 75^th^ percentiles for BL were reported in original paper. Mean BL estimated as median BL. SD for BL estimated assuming distribution is normal as SD = Abs[(75th percentile-25th percentile)/(Z(.75)-Z(.25))]. |
| Edwards 2021 |  | 65.00 | -1.50 | 13.04 | -0.12 | 0 | 0 | NA | |  | Mean difference in LVMI ∆ reported in original paper |

Abbreviations: ∆ = change; Abs = absolute value; ACE = angiotensin converting enzyme; ACM = all-cause mortality; ADPKD = autosomal dominant polycystic kidney disease; ARB = angiotensin receptor blocker; BMI = body mass index; BL = baseline; BNP = b-type natriuretic peptide; BP = blood pressure; CAPD = continuous ambulatory peritoneal dialysis; CKD = chronic kidney disease; CrCl = Creatinine clearance; CVM = cardiovascular mortality; cMRI = cardiac magnetic resonance imaging; DM = diabetes mellitus; eGFR = estimated glomerular filtration rate; EOS = end of study; ESA = erythropoietin stimulating agent; ESKD = end-stage kidney disease; F/U = follow-up; GRF = glomerular filtration rate; Hb = hemoglobin; HD = hemodialysis; HDF = hemodiafiltration; hGH = .human growth hormone; HTN = hypertension; LVEF = left ventricular ejection fraction; LVM = left ventricular mass; LVMI = left ventricular mass index; LVVI = left ventricular volume index; LVH = left ventricular hypertrophy; MRI = magnetic resonance imaging; PD = peritoneal dialysis; PTH = parathyroid hormone; RAASi = renin-angiotensin-aldosterone system inhibitor; RND = randomized; SEM = standard error of the mean; SD = standard deviation; sHPT = secondary hyperparathyroidism; SMD = standardized mean difference; Stnd tx = standard treatment; Tx = treatment

Italics represent mean level of baseline kidney function in studies involving non-dialysis CKD patients.

# Table S3: Risk of bias assessment including studies with ≥6 months of follow-up that observed a reduction in LVM/LVMI

| **Study** | **Random**  **sequence generation** | **Allocation concealment** | **Blinding of participants** | **Blinding of investigators** | **Blinding of outcome assessors** | **Incomplete data reporting** | | **Selective outcome reporting** |
| --- | --- | --- | --- | --- | --- | --- | --- | --- |
| ***Higher hemoglobin target using erythropoiesis-stimulating agents (ESA)*** | | | | | |  | |  |
| Levin, 2005 | Low risk | Low risk | High risk | High risk | Low risk | Low risk | | Low risk |
| Parfrey, 2005 | Low risk | Low risk | Low risk | Low risk | Low risk | Low risk | | Low risk |
| Macdougall, 2007 | Unclear risk | Low risk | High risk | High risk | Unclear risk | Unclear risk | | Low risk |
| Ritz, 2007 | Low risk | Low risk | High risk | High risk | Low risk | Low risk | | Unclear risk |
| Pappas, 2008 | Unclear risk | Unclear risk | High risk | High risk | Low risk | Unclear risk | | Unclear risk |
| Cianciaruso, 2008 | Low risk | Low risk | High risk | High risk | Low risk | Low risk | | Low risk |
| Akizawa, 2011 | Low risk | Unclear risk | High risk | High risk | Unclear risk | Unclear risk | | Unclear risk |
| ***RAAS inhibitors*** | | | | | | | | |
| Suzuki, 2002 | Unclear risk | Unclear risk | High risk | High risk | Unclear risk | Unclear risk | | Unclear risk |
| Suzuki, 2003 | Unclear risk | Unclear risk | Low risk | Low risk | Unclear risk | Unclear risk | | Unclear risk |
| Kanno, 2004 | Unclear risk | Unclear risk | Low risk | Low risk | Unclear risk | Low risk | | Unclear risk |
| Matsumoto, 2006 | Unclear risk | Unclear risk | Low risk | Low risk | Unclear risk | Unclear risk | | Unclear risk |
| Yu, 2006 | Unclear risk | Unclear risk | Low risk | Low risk | Unclear risk | Unclear risk | | Unclear risk |
| Mitsuhashi, 2009 | Unclear risk | Unclear risk | High risk | High risk | Unclear risk | Unclear risk | | Unclear risk |
| Taheri, 2009 | Unclear risk | Unclear risk | Low risk | Low risk | Low risk | Low risk | | Unclear risk |
| Ito, 2014 | Unclear risk | Unclear risk | High risk | High risk | Low risk | Low risk | | Low risk |
| Feniman-De-  Stefano 2015 | Unclear risk | Unclear risk | Low risk | Low risk | Unclear risk | Low risk | | Low risk |
| London, 1994 | Low risk | Unclear risk | Low risk | Low risk | Low risk | Low risk | | Unclear risk |
| Shibasaki, 2002 | Unclear risk | Unclear risk | Low risk | Low risk | Low risk | Unclear risk | | Unclear risk |
| Zeltner, 2008 | Unclear risk | Unclear risk | Low risk | Low risk | Unclear risk | Low risk | | Unclear risk |
| Yilmaz, 2010 | Unclear risk | Unclear risk | High risk | High risk | Unclear risk | Low risk | | Unclear risk |
| Ulusoy, 2010 | Unclear risk | Unclear risk | High risk | High risk | Unclear risk | Unclear risk | | Unclear risk |
| ***Hemodialysis therapy*** | | | | | | | | |
| Schrander-vd Meer, 1999 | Unclear risk | Unclear risk | High risk | High risk | Low risk | Low risk | | Unclear risk |
| Alvestrand, 2011 | Low risk | Unclear risk | High risk | High risk | Low risk | Low risk | | Low risk |
| Ohtake, 2012 | Low risk | Unclear risk | High risk | High risk | Low risk | Unclear risk | | Unclear risk |
| Maduell, 2012 | Unclear risk | Unclear risk | High risk | High risk | Low risk | Unclear risk | | Unclear risk |
| Mostovaya, 2014 | Low risk | Unclear risk | High risk | High risk | Low risk | Unclear risk | | Unclear risk |
| Culleton, 2007 | Low risk | Low risk | High risk | High risk | Low risk | Low risk | | Low risk |
| Katopodis, 2009 | High risk | High risk | High risk | High risk | Unclear risk | Unclear risk | | Unclear risk |
| Chertow, 2010 | Low risk | Low risk | High risk | High risk | Low risk | Low risk | | Low risk |
| Rocco, 2011 | Low risk | Low risk | High risk | High risk | Low risk | Low risk | | Low risk |
| Jardine, 2017 | Low risk | Low risk | High risk | High risk | Unclear risk | Unclear risk | | Low risk |
| Liu, 2014 | Unclear risk | Unclear risk | High risk | High risk | Unclear risk | Low risk | | Low risk |
| Hur, 2013 | Unclear risk | Unclear risk | High risk | High risk | Low risk | Low risk | | Low risk |
| Chen, 2011 | Low risk | Unclear risk | High risk | High risk | Unclear risk | Low risk | Low risk | |
| Whalley, 2013 | Low risk | Low risk | High risk | High risk | Low risk | Low risk | Low risk | |
| Odudu 2015 | Low risk | Low risk | High risk | High risk | Low risk | Low risk | Low risk | |
| Liu, 2016 | Low risk | Unclear risk | Unclear risk | Unclear risk | Unclear risk | Low risk | | Low risk |
| Marshall 2020 | Low risk | Low risk | Unclear risk | Unclear risk | Low risk | Low risk | Low risk | |
| ***Other interventions*** | | | | | | | | |
| Ivarsen, 2012 | Low risk | Low risk | High risk | High risk | Low risk | Low risk | | Unclear risk |
| Dreyer, 2014 | Low risk | Low risk | Low risk | Low risk | Low risk | Low risk | | Low risk |
| Howden, 2013 | Low risk | Unclear risk | High risk | High risk | Low risk | Low risk | | Low risk |
| Schrier, 2002 | Low risk | Unclear risk | High risk | High risk | Low risk | Low risk | | Low risk |
| Li, 2011 | Low risk | Unclear risk | High risk | High risk | Unclear risk | Low risk | | Low risk |
| Li, 2013 | Low risk | Unclear risk | High risk | High risk | Unclear risk | Low risk | | Low risk |
| Feldt-Rasmussen, 2007 | Low risk | Low risk | Low risk | Low risk | Low risk | Low risk | | Low risk |
| Nakamura, 2002 | Unclear risk | Unclear risk | Low risk | Low risk | Unclear risk | Low risk | | Unclear risk |
| Hotu, 2010 | Unclear risk | Unclear risk | High risk | High risk | Low risk | Low risk | | Low risk |
| Kao, 2011 | Unclear risk | Unclear risk | Low risk | Low risk | Unclear risk | Low risk | | Low risk |
| Zamboli, 2011 | Low risk | Low risk | High risk | High risk | Low risk | Low risk | | Low risk |
| Higuchi 2016 | Low risk | Unclear risk | High risk | High risk | Low risk | Low risk | | Low risk |
| Lin, 2016 | Low risk | Low risk | Low risk | Low risk | Unclear risk | Low risk | | Unclear risk |
| Charytan 2019 | Low risk | Low risk | Low risk | Low risk | Low risk | Low risk | | Low risk |
| Miskulin 2018 | Low risk | Low risk | High risk | High risk | High risk | Low risk | | Low risk |
| Djuric 2020 | Low risk | Unclear risk | Low risk | Low risk | Low risk | Low risk | | Unclear risk |
| Fujii 2018 | Low risk | Unclear risk | High risk | High risk | Low risk | Low risk | | Unclear risk |
| Rutherford 2021 | Low risk | Unclear risk | Low risk | Low risk | Low risk | Low risk | | Unclear risk |
| Dorr 2021 | Low risk | Unclear risk | Low risk | Unclear risk | Low risk | Low risk | | Unclear risk |
| Edward 2021 | Low risk | Unclear risk | High risk | High risk | Low risk | Low risk | | Low risk |

# Table S4. Assessment of the quality of evidence using the GRADE approach

| **Number of**  **trials/participants** | **Within-study risk of bias** | **Indirectness** | **Heterogeneity** | **Imprecision** | **Quality of evidence** |  |
| --- | --- | --- | --- | --- | --- | --- |
| **Higher hemoglobin target using ESA vs. lower hemoglobin target** | |  |  |  |  |  |
| 7/1585 | No serious limitations: allocation concealment- unclear risk in 2 (29%) trials, blinding of outcome assessors- unclear risk in 2 (29%) trials, incomplete data- unclear risk in 3 (43%) trials, selective outcome reporting- unclear risk in 3 (43%) trials. Blinding of participants and investigators- high risk in 6 (86%) trials. | Direct | No important heterogeneity;  I^2^ 0% | Imprecise (-1) | Moderate |  |
| **RAAS inhibitors** | |  |  |  |  |  |
| 14/623 | Potential limitations (-1): allocation concealment- unclear risk in 14 (100%) trials, blinding of outcome assessors- unclear risk in 10 (71%) trials, incomplete data- unclear risk in 7 (50%) trials, selective outcome reporting- unclear risk in 12 (86%) trials. Blinding of participants and investigators- high risk in 5 (36%) trials. | Direct | No important heterogeneity;  I^2^ 0% | Imprecise (-1) | Low to Moderate |  |
| **Hemodialysis** | |  |  |  |  |  |
| 17/2166 | Potential limitations (-1): allocation concealment- unclear risk in  9 (53%) trials and high risk in 1 (6%) trial, blinding of outcome assessors- unclear risk in 5 (29%) trials, incomplete data- unclear risk in 5 (29%) trials, selective outcome reporting- unclear risk in 5 (29%) trials. Blinding of participants and investigators- high risk in 15 (88%) trials and unclear risk in 2 (12%) trials. Data on adverse events was not reported in 3 (18%) trials. Low study completion rate in 1 (6%) trial. | Direct | No important heterogeneity;  I^2^ 0% | Imprecise (-1) | Low to Moderate |  |
| **Other** | | | | | | |
| 20/1913 | Potential limitations (-1): allocation concealment- unclear risk in 13 (65%) trials, blinding of outcome assessors- unclear or high risk in 6 (30%) trials, selective outcome reporting- unclear risk in 7 (35%) trials. Blinding of participants and investigators- high risk in 11 (55%) trials. | Direct | No important heterogeneity;  I^2^ 0% | Imprecise (-1) | Low to Moderate |  |

GRADE= Grading of Recommendations Assessment, Development and Evaluation; ESA= erythropoiesis-stimulating agent; RAAS= renin-angiotensin-aldosterone system.
